# Genomic architecture of potato resistance to *Synchytrium endobioticum* disentangled using SSR markers and the 8.3k SolCAP SNP genotyping array

**DOI:** 10.1186/s12863-015-0195-y

**Published:** 2015-04-16

**Authors:** Jude Eijkeme Obidiegwu, Rena Sanetomo, Kerstin Flath, Eckhard Tacke, Hans-Reinhard Hofferbert, Andrea Hofmann, Birgit Walkemeier, Christiane Gebhardt

**Affiliations:** Department of Plant Breeding and Genetics, Max-Planck-Institute for Plant Breeding Research, Cologne, Germany; National Root Crops Research Institute Umudike, PMB 7006, Umuahia, Abia State Nigeria; Obihiro University of Agriculture and Veterinary Medicine, Obihiro, Hokkaido 080-8555 Japan; Julius Kühn Institute, Federal Research Centre for Cultivated Plants, Kleinmachnow, Germany; Bioplant GmbH, Ebstorf, Germany; Böhm-Nordkartoffel Agrarproduktion GbR, Ebstorf, Germany; Department of Genomics, Life & Brain Center, Institute of Human Genetics, University of Bonn, Bonn, Germany

**Keywords:** *Synchytrium endobioticum*, Potato wart, DNA-based marker, Linkage mapping, Resistance breeding, Bulked segregant analysis (BSA), Single nucleotide polymorphism (SNP), Genotyping array, Quantitative resistance

## Abstract

**Background:**

The soil borne, obligate biotrophic fungus *Synchytrium endobioticum* causes tumor-like tissue proliferation (wart) in potato tubers and thereby considerable crop damage. Chemical control is not effective and unfriendly to the environment. *S. endobioticum* is therefore a quarantined pathogen. The emergence of new pathotypes of the fungus aggravate this agricultural problem. The best control of wart disease is the cultivation of resistant varieties. Phenotypic screening for resistant cultivars is however time, labor and material intensive. Breeding for resistance would therefore greatly benefit from diagnostic DNA markers that can be applied early in the breeding cycle. The prerequisite for the development of diagnostic DNA markers is the genetic dissection of the factors that control resistance to *S. endobioticum* in various genetic backgrounds of potato.

**Results:**

Progeny of a cross between a wart resistant and a susceptible tetraploid breeding clone was evaluated for resistance to *S. endobioticum* pathotypes 1, 2, 6 and 18 most relevant in Europe. The same progeny was genotyped with 195 microsatellite and 8303 single nucleotide polymorphism (SNP) markers. Linkage analysis identified the multi-allelic locus *Sen1/RSe-XIa* on potato chromosome XI as major factor for resistance to all four *S. endobioticum* pathotypes*.* Six additional, independent modifier loci had smaller effects on wart resistance. Combinations of markers linked to *Sen1/RSe-XIa* resistance alleles with one to two additional markers were sufficient for obtaining high levels of resistance to *S. endobioticum* pathotypes 1, 2, 6 and 18 in the analyzed genetic background.

**Conclusions:**

Potato resistance to *S. endobioticum* is oligogenic with one major and several minor resistance loci. It is composed of multiple alleles for resistance and susceptibility that originate from multiple sources. The genetics of resistance to *S. endobioticum* varies therefore between different genetic backgrounds. The DNA markers described in this paper are the starting point for pedigree based selection of cultivars with high levels of resistance to *S. endobioticum* pathotypes 1, 2, 6 and 18.

**Electronic supplementary material:**

The online version of this article (doi:10.1186/s12863-015-0195-y) contains supplementary material, which is available to authorized users.

## Background

*Synchytrium endobioticum* (Schilberszky) Percival is a soil borne, obligate biotrophic fungus of the order Chytridiales in the phylum Chytridiomycota, which infects tubers of the potato (*Solanum tuberosum* L.) causing the wart disease. The fungus induces cell divisions in the tuber which proliferate into tumor-like tissues at the tuber’s expense. The wart tissues finally decompose and release resting sporangia (sori) which remain viable for more than 30 years in the soil. Chemical control measures are not effective. S. *endobioticum* is therefore a quarantined pathogen and infested fields are forbidden for potato cultivation for many years. This fact is the major reason for economic losses due to S. *endobioticum* infestation, besides yield losses directly connected with the disease symptoms. Occurrence of *S. endobioticum* has been recorded worldwide. However cool, humid temperate zones and intensive potato cultivation are favorable environments for the pathogen [1]. The only sustainable solution to this problem is the cultivation of wart resistant varieties. Classical breeding for resistance began around 100 years ago and was highly successful in selecting cultivars resistant to *S. endobioticum* pathotype 1, the most common pathotype in Europe during the first half of the 20^th^ century. Resistance was found in varieties such as ‘Snowdrop’ and ‘Flourball’ and in a number of wild tuber bearing species such as *Solanum acaule* [2,3]. Resistant varieties and strict quarantine measures curtailed the wart disease so effectively, that breeding for wart resistance lost priority until new pathotypes of *S. endobioticum* appeared, against which the widely distributed resistance against pathotype 1 was not effective any more. Increasing potato trade within Europe also increases the risk of wart dissemination via adhering soil contaminated with sori [1]. There is a renewed necessity therefore to develop varieties which combine good agronomic qualities with resistance against the currently most important *S. endobioticum* pathotypes 1, 2, 6 and 18 [4]. Wart resistance assessment is labor and time intensive due to the obligate biotrophic lifestyle of *S. endobioticum* and the time needed for warts to develop (3-4 weeks) [5]. Moreover the evaluation requires at least ten tubers per genotype and pathotype, which become available only after several rounds of multiplication. Replacing phenotypic screening in the early stages of the breeding cycle by DNA-based markers that are closely linked or even identical with genes for resistance to *S. endobioticum* would greatly enhance the efficiency and precision of identifying wart resistant cultivars. The prerequisite for the identification of such markers is the genetic dissection of potato resistance against the different *S. endobioticum* pathotypes based on genome wide linkage or association mapping with molecular markers.

The first locus conferring resistance to *S. endobioticum* pathotype 1 (*Sen1*) was identified on the distal end of the long arm of potato chromosome XI based on restriction fragment length polymorphism (RFLP) linkage mapping in a diploid F1 family [6]. *Sen1* is part of a ‘hot spot’ for qualitative and quantitative resistance against viruses, bacteria, fungi and nematodes in the potato genome [7-15]. Some or all of these resistance genes including *Sen1,* might be encoded by a large cluster of approximately twenty NB-LRR (nucleotide binding-leucine rich repeat) type genes located in the distal 10 Mega base pairs (Mbp) of the long arm of chromosome XI, some of which share high sequence similarity with the tobacco *N* gene for resistance to *Tobacco Mosaic Virus* (TMV) [6,16,17]. None of these NB-LRR type genes except *Y-1* [18] has been functionally characterized. A length polymorphism in the *N*-like gene *Nl25* was diagnostic for *Sen1* in progeny of the diploid resistant parent of the original mapping population [19] but this polymorphism was not detectable in tetraploid cultivars (unpublished observation). The second locus for resistance to *S. endobioticum* pathotype 1 (*Sen1-4*) was genetically mapped on the long arm of chromosome IV approximately 5 cM proximal to the centromere [20]. In this case amplified fragment length polymorphism (AFLP) markers and a diploid F1 family unrelated to the one characterized by Hehl et al. [6] were used for mapping. Physical mapping placed *Sen1-4* in a BAC (bacterial artificial chromosome) contig of approximately 1 Mbp [20]. More recently, three tetraploid F1 families, two of them half sib families, were phenotyped for resistance to wart and genotyped with simple sequence repeat (SSR), single nucleotide polymorphism (SNP) and/or AFLP markers. These families segregated for resistance to *S. endobioticum* pathotypes 1, 2, 6 and 18 (P1, P2, P6, P18) inherited from different resistant parents [5,21]. Resistance to all four pathotypes showed a quantitative phenotypic distribution in the three families, which is in contrast to the diploid families, where resistance to *S. endobioticum* pathotype1 segregated as a single dominant gene. In both studies, a major quantitative resistance locus (QRL) conferring resistance to pathotype 1 was detected on chromosome XI, closely linked or identical with the *Sen1* locus. Due to lack of common markers used in both studies, it is unclear to what extent the resistance alleles segregating in the three families were the same. Common to both studies was also the fact that resistance to P2, P6 and P18 was highly correlated. Accordingly, QRL against pathotypes P2, P6 and P18 were detected via linkage with the same markers. However the QRL mapped to different chromosomes, to chromosome I in Ballvora et al. [5], and to chromosomes II, VI, VII, VIII and X in Groth et al. [21]. Besides *Sen1*, additional QRL against P1 were mapped to chromosomes II, VI and VIII, in the same regions as the QRL against P2, P6 and P18 [21]. The molecular genetic studies on the inheritance of wart resistance available to date [5,6,20,21] collectively indicate that the genetic architecture of resistance to *S. endobioticum* depends on the ploidy level and the specific genetic background used for mapping, with respect to both resistant as well as susceptible parents.

Neither RFLP nor AFLP markers are practicable for high throughput screening in breeding programs. The markers of choice today are SSRs, SNPs and polymerase chain reaction (PCR) assays that tag directly and specifically a particular trait allele. The limited number of potato SSR markers [22-24] might be insufficient to capture all resistance alleles present in a given genetic background. SNPs linked to wart resistance alleles are restricted so far to three QRL described in Ballvora et al. [5]. Now genome coverage can be enhanced from few hundred to several thousand markers via genotyping with the 8.3k potato SNP genotyping array [25,26]. This improves the chances to detect comprehensively all loci that contribute to wart resistance in a particular genetic background. Moreover the potato genome sequence [27] and improved physical map [28] allow the genomic dissection of wart resistance loci via physical mapping of QRL linked DNA markers.

In the present study, we analyzed a new family of tetraploid potato genotypes with a genetic background different from previous studies for the inheritance of wart resistance to pathotypes 1, 2, 6 and 18. Genotyping this family with SSRs and the 8.3k SolCAP SNP genotyping array identified novel as well as known loci for resistance to *S. endobioticum*. The results are integrated with previous molecular linkage maps via the potato reference genome sequence [27,28] in order to obtain a comprehensive view on the genomic architecture of resistance to wart.

## Results

### Phenotypic evaluation of wart resistance

**Figure 1:**
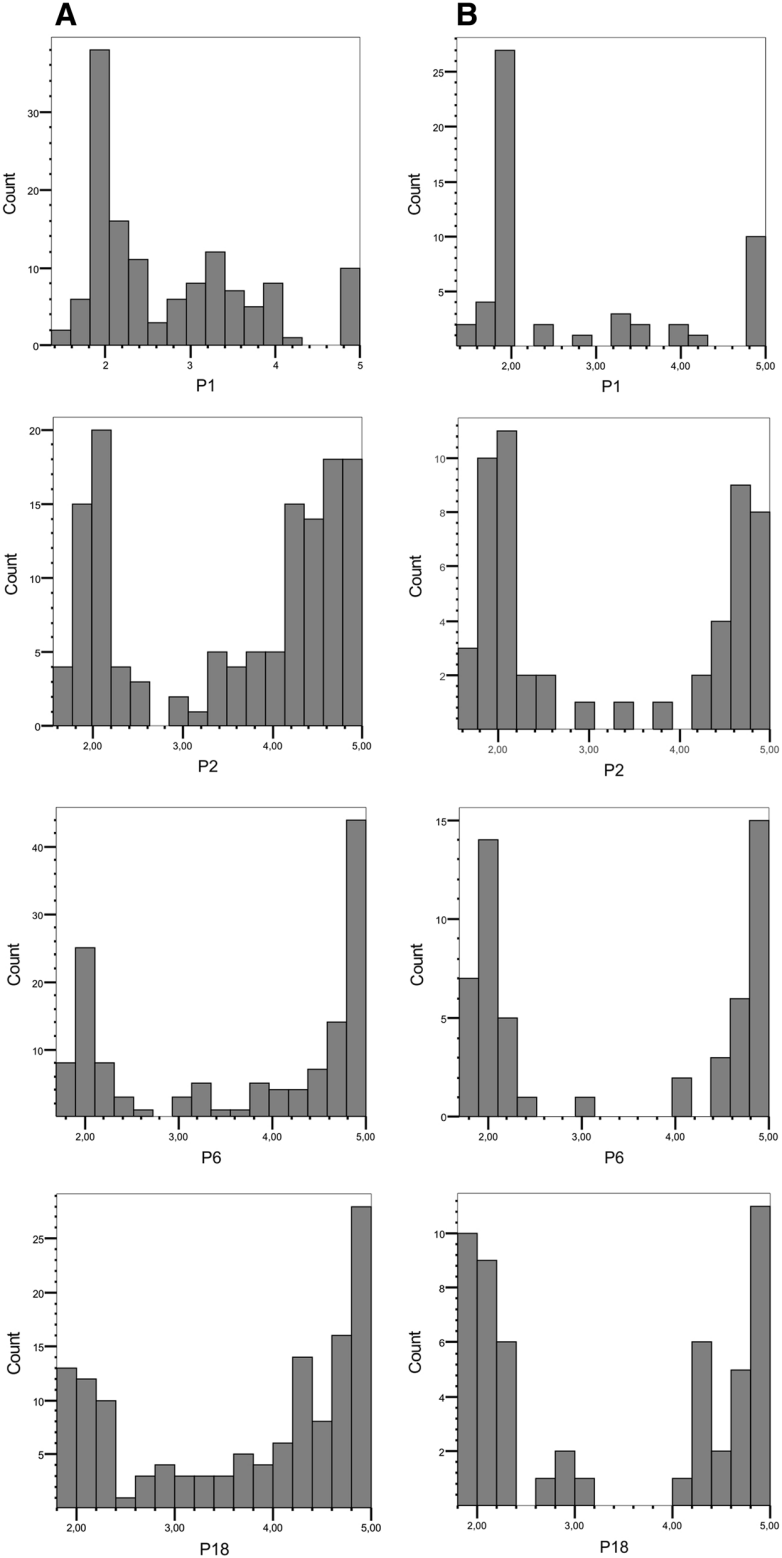
Histograms of the mean scores for resistance to *S. endobioticum* pathotype 1 (P1), 2 (P2), 6 (P6) and 18 (P18). **(A)** Histograms of the full BNA2 family (n = 133) and **(B)** of the selected subset of 54 genotypes used for genome wide SNP genotyping.

The scores for resistance to *S. endobioticum* pathotypes P1, P2, P6 and P18 in the BNA2 family (n = 133) showed for all pathotypes a bi-phasic phenotypic distribution. The minimum between the two peaks was between scores 2.5 and 2.7 (Figure 1A). When adopting the mean score 2.49 as cut-off for resistance, 71 genotypes were resistant to P1 and 62 were susceptible. This distribution fitted the model of a single dominant gene for resistance present in simplex dosage in the resistant parent Ps-355 and inherited with a 1 : 1 segregation ratio (χ^2^ = 0.61, p > 0.05). However, only 16 genotypes were fully susceptible (score 4.00-5.00), whereas 46 genotypes scored intermediate between 2.50 and 3.99. The ‘susceptible’ parent Ps-354 was also moderately resistant to P1 (mean score 2.35). This indicated that additional factors for resistance to P1 segregated in the BNA2 family, which inherited also from parent Ps-354. Using the same cut-off value, resistance to P2, P6 and P18 segregated 45 to 88 (P2), 43 to 90 (P6) and 36 resistant to 97 susceptible genotypes (P18). The segregation ratios skewed toward susceptibility and the presence of genotypes with intermediate resistance scores suggested the presence of a major factor for resistance to P2, P6 and P18, which was suppressed or modified by additional genes. Resistance to P2, P6 and P18 was highly correlated, suggesting that resistance to pathotypes P2, P6 and P18 was conferred by the same or tightly linked factors. The correlation between resistance to P1 and P2, P6, P18 was less strong but still highly significant (Table 1).

**Table 1:** **Pearson’s coefficient of correlation between resistance to** ***S. endobioticum*** **pathotypes 1, 2, 6 and 18**

	**Pathotype 1**	**Pathotype 2**	**Pathotype 6**
Pathotype 2	0.592***^a^		
Pathotype 6	0.604***	0.927***	
Pathotype 18	0.623***	0.882***	0.857***

^a^***indicates significance of the correlation at p < 0.001.

### Bulked segregant analysis with SSR markers

**Figure 2:**
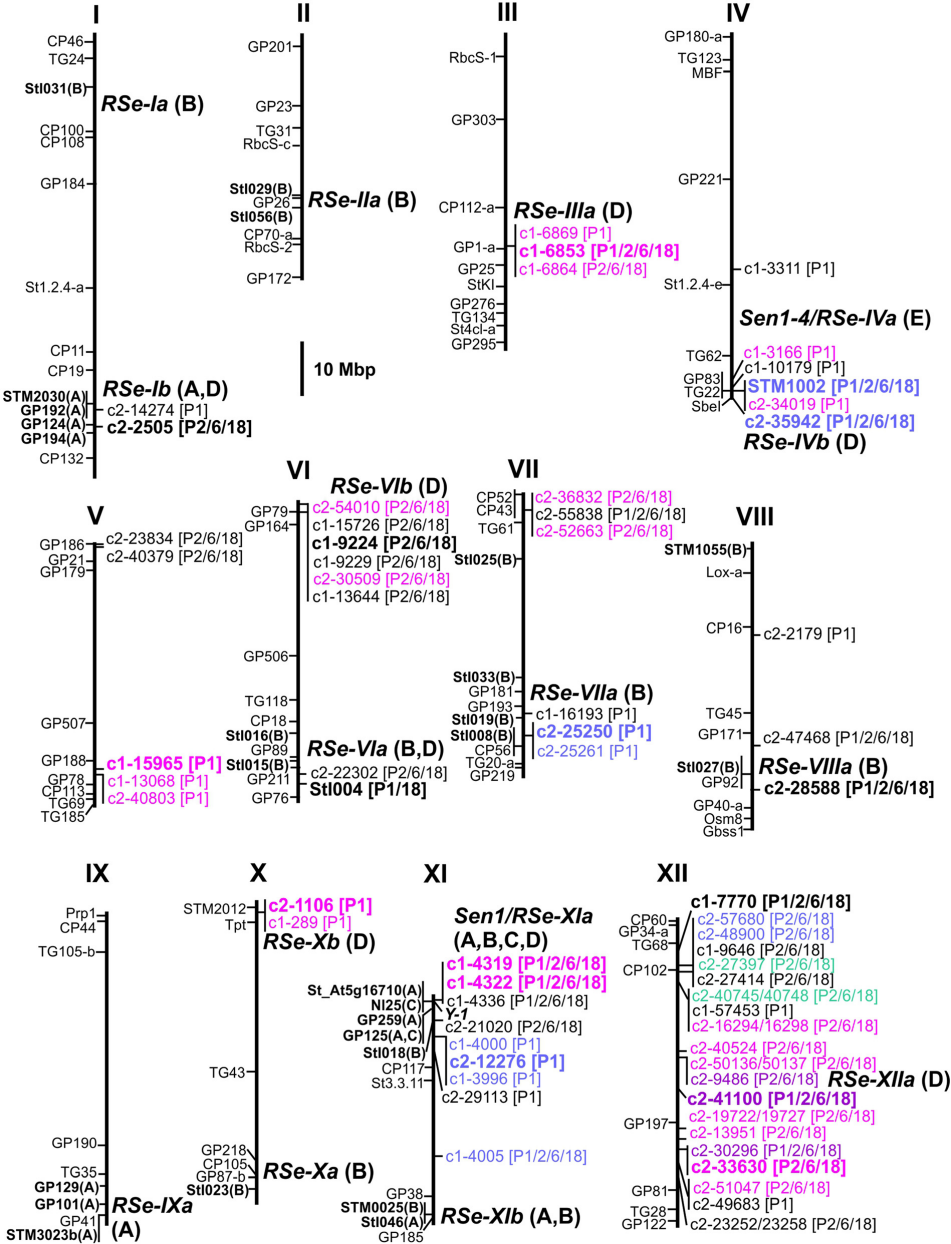
Physical map of the twelve potato chromosomes (pseudomolecules v4.03) [28] for wart resistance loci. The positions of markers linked with wart resistance loci in previous studies are shown on the left in **bold letters**. Capital letters in parenthesis are codes for the corresponding reference: **A** [5], **B** [21] and C [6]. For general orientation, additional sequence based markers of potato and tomato are shown on the left (not bold), which anchor potato genetic maps (http://www.gabipd.org/database/maps.shtml) to the physical map. The positions of SSR markers STM1002, StI004 and the SolCAP SNPs linked to wart resistance loci in the subset of 54 BNA2 genotypes are shown on the right, with pathotype range in brackets. Markers which co-segregated in coupling phase or were tightly linked and had the same parental allele configuration (see Additional file 2) are shown in the same color. Markers that were tested in the full BNA2 family (n = 133) are in **bold letters**. The approximate positions of *RSe* loci known to date are indicated on the right with the corresponding reference code in parenthesis: **A** [5], **B** [21], **C** [6], **D** (this paper) and **E** [20].

Resistant and susceptible DNA bulks composed of twelve F1 genotypes each and the parents Pr-355 and Ps-354 of the BNA2 family were screened with 195 SSR markers, including markers STM2030 (chromosome I), STM3023b (chromosome IX) and StI046 (chromosome XI) that have been shown to be linked to wart QRL in the previously analyzed BNA1 and SaKa1 families [5]. This resulted in twelve candidate SSR markers, two of which could be subsequently confirmed to be linked with wart QRL in the BNA2 family (Table 2). The SSR markers STM1002 and StI004 (see Additional file 1) were located in distal positions on the long arm of chromosomes IV and VI, respectively (Figure 2). The alleles *STM1002-224* and *StI004-96* were linked with wart resistance alleles descended from the resistant parent Pr-355. *STM1002-224* was linked with a QRL for all four pathotypes, whereas *StI004-96* was linked with a QRL against P1 and P18 (Table 2).

**Table 2:** **Markers linked with resistance to** ***S. endobioticum*** **pathotypes 1, 2, 6 and 18 in the BNA2 family**

**Marker**	**Chr. no.**	**Alleles** ^**e**^	**Parental genotype Ps-354**	**Parental genotype Pr-355**	**P1, χ** ^**2g**^	**P2, χ** ^**2**^	**P6, χ** ^**2**^	**P18, χ** ^**2**^
Solcap_c2_2505	I	*A↑/G*	*GAAA*	*GGGA*	16.27**^f^	ns^f^	ns	ns
GP194_snp7^b^	I	*A/T↑*	*AATT*	*AAAT*	ns	ns	ns	8.09*
Solcap_c1_6853	III	*T/G↑*	*TTTT*	*TTTG*	ns	7.75*	8.91*	ns
STM1002-224^a^	IV	*1↑/0*	*0*	*1*	7.93**	8.03**	8.99**	8.20**
Solcap_c2_35942	IV	*A↑/G*	*GGGG*	*AGGG*	14.99***	11.28**	12.78 ***	10.62**
Solcap_c1_15965	V	*T/G*	*TTTG*	*TGGG*	ns	ns	ns	ns
StI004-96^a^	VI	*1↑/0*	*0*	*1*	9.25**	ns	ns	11.04**
Solcap_c1_9224	VI	*T/C↑*	*TCCC*	*CCCC*	ns	ns	ns	8.28*
Solcap_c2_25250	VII	*T/C*	*TTTC*	*TTTC*	ns	ns	ns	ns
Solcap_c2_28588	VIII	*T/G*	*TTTT*	*TTTG*	ns	ns	ns	ns
Solcap_c2_1106	X	*G↑/C*	*CCCC*	*GCCC*	15.26***	13.34***	8.45 **	13.76***
Solcap_c1_4319^c^	XI	*A↑/G*	*AGGG*	*AGGG*	23.25***	32.77***	38.70***	21.16***
Solcap_c1_4319_2	XI	*G/A*	*GGAA*	*AAAA*	ns	ns	ns	ns
Solcap_c1_4319_3	XI	*T/C*	*TCCC*	*CCCC*	ns	ns	ns	ns
Solcap_c1_4322^c^	XI	*T↑/C*	*TCCC*	*TCCC*	18.40***	34.55***	40.20***	18.86***
Y1delATT	XI	*1↑/0*	0	1	32.70***	51.05***	46.59***	46.11***
Solcap_c2_12276	XI	*A/C*	*ACCC*	*ACCC*	ns	ns	ns	ns
GP125_snp10^b^	XI	*C↑/A*	*CCCC*	*CCCA*	7.58**	5.48*	6.16*	ns
GP259_snp7^b^	XI	*A/G↑*	*AAAG*	*AAAG*	10.18*	18.58***	13.17**	20.72***
GP259_snp2^b^	XI	*G↑/A*	*GGGG*	*GGAA*	6.84*	ns	ns	7.94*
GP259_snp16/17^b^	XI	*AC/GA↑*	*AAAG/CCCA*	*AAAA/CCCC*	ns	7.57 **	15.31***	ns
St_At5g16710_snp4^b^	XI	*G/C↑*	*GGGC*	*GGGG*	ns	14.26**	17.21**	8.16*
St_At5g16710_snp5^b^	XI	*A↑/G*	*AAGG*	*AAAA*	ns	7.04*	ns	ns
St_At5g16710_snp1^b,d^	XI	*T/A↑*	*TTAA*	*TAAA*	ns	14.21**	13.61**	9.46*
St_At5g16710_snp3^b,d^	XI	*C/A↑*	*CCAA*	*CAAA*	ns	14.47**	14.82**	10.42*
St_At5g16710_snp6^b,d^	XI	*A/T↑*	*AATT*	*ATTT*	ns	15.73**	12.76**	9.01*
St_At5g16710_snp10^b^	XI	*A/G↑*	*AGGG*	*AAAG*	10.40*	ns	ns	ns
St_At5g16710_snp11^b^	XI	*C↑/T*	*CCCT*	*CCTT*	14.75**	8.50*	11.84**	10.10*
Solcap_c1_7770	XII	*T/C↑*	*TTTC*	*TTCC*	ns	12.20**	8.15*	18.64***
Solcap_c2_33630	XII	*T/C↑*	*TTCC*	*TTCC*	ns	17.75**	18.41**	15.81**
Solcap_c2_33630_1 (~Solcap_c2_41100)	XII	*A/G↑*	*AAGG*	?	ns	17.76**	17.64**	21.41**
Solcap_c2_33630_2	XII	*G/A↑*	*GGAA*	*GGAA*	9.74*	21.72***	19.27**	23.03***

^a^Marker was genotyped in 94 BNA2 individuals.

^b^Marker was genotyped in 90 BNA2 individuals.

^c^SNPs c1_4319 and c1_4322 co-segregated in the BNA2 family.

^d^SNPs St_At5g16710_snp1, St_At5g16710_snp3 and St_At5g16710_snp6 co-segregated in the BNA2 family.

^e^The allele linked to greater resistance is indicated by *↑*.

^f^ns: p > 0.05, *0.05 > p > 0.01, **0.01 > p > 0.001, ***p < 0.001.

^g^Chi-square values were obtained with the Kruskal-Wallis test for a phenotypic difference between genotypic classes.

### Genome wide genotyping with the 8.3k SolCAP potato SNP array

Fifty four genotypes were selected from the BNA2 family based on the resistance scores. Most genotypes with intermediate resistance levels were removed which led to a more pronounced bi-phasic phenotypic distribution for pathotypes P1, P2, P6 and P18 (Figure 1B). Genotyping the parents and 54 F1 progeny with the 8.3k SolCAP potato SNP array resulted in 6286 polymorphic SNPs. Sixty seven SNPs with unambiguous genomic positions showed linkage (*p* < 0.01) with QRL, either for P1 only or for P2, P6 and P18 or for all pathotypes (see Additional file 2 and Figure 2). Forty of the 67 SNPs either co-segregated or were tightly linked in coupling phase with one or more other SNPs and had the same parental allele dosage. Most prominent in this respect was chromosome XII, where 18 SNPs distributed over the central 40 Mbp fell into four groups of genetically tightly linked SNP markers with the parental allele dosages simplex/simplex, duplex/nulliplex, simplex/duplex and duplex/duplex (see Additional file 2 and Figure 2). A second group of four co-segregating SNPs (parental allele dosage simplex/simplex) covered the central 24 Mbp of chromosome XI. The remaining genetically tightly linked SNPs were physically linked within two Mbp or less (Figure 2). The 67 SNPs clustered in thirteen genomic segments on ten potato chromosomes. Among those were the two segments harboring the SSR markers STM1002 and StI004 (Figure 2). Most significantly linked (*p* ≤ 0.001) with resistance against all pathotypes were the SolCAP SNPs c1_4319 and c1_4322 on the distal end of chromosome XI and c1_7770 on chromosome XII. Three SNPs on chromosome XII (c2_40745, c2_41100) and VI (c1_9224) were linked at *p* < 0.001 with resistance loci against P2, P6 and P18 (see Additional file 2).

### SNP markers linked with wart resistance loci in the BNA2 family

**Figure 3:**
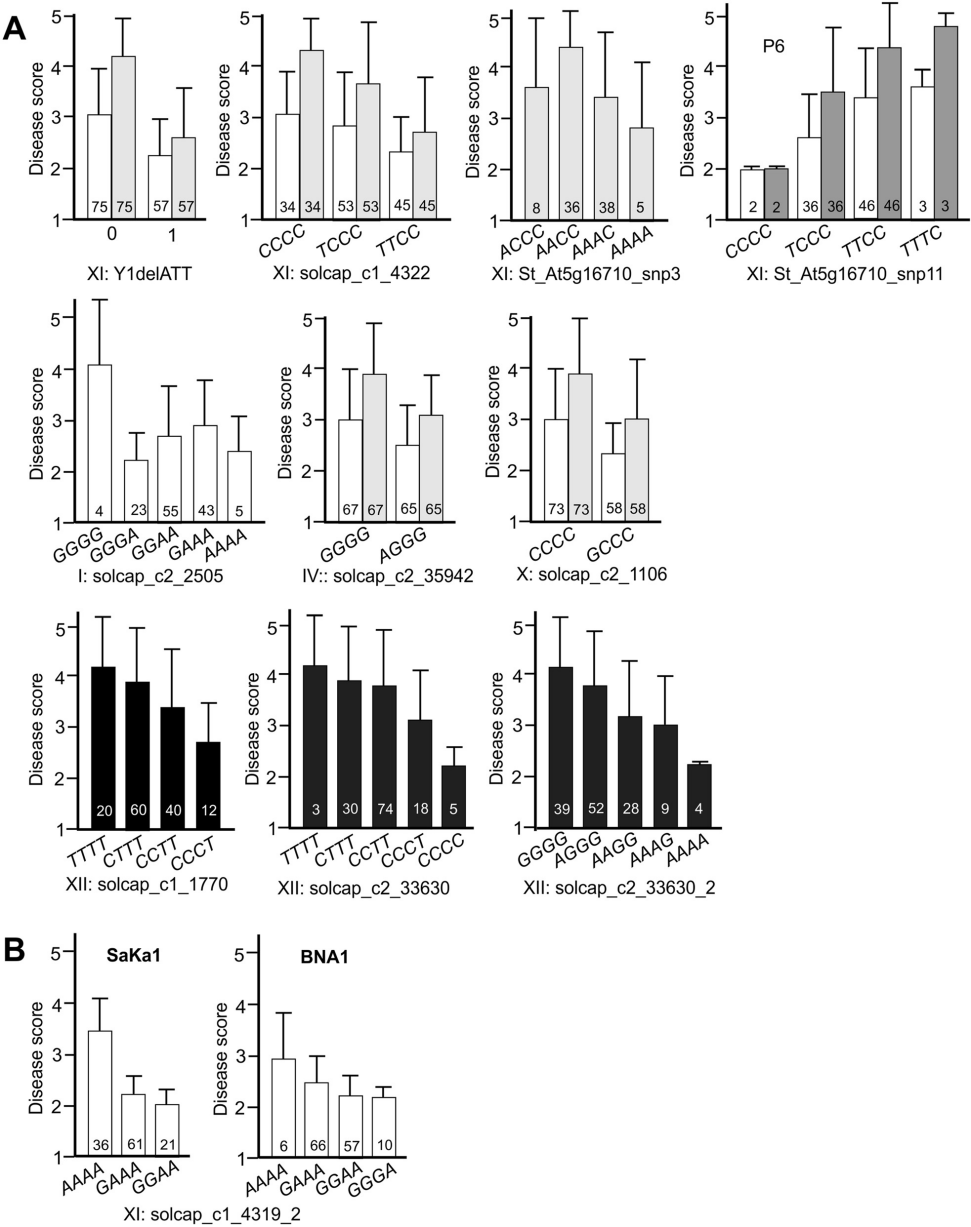
Effects of single markers on mean wart resistance. **A**: Effect of presence/absence (1/0) of Y1delATT and allele dosage of nine SNPs on resistance to P1 (white bars), P2 (light grey bars), P6 (dark grey bars) and P18 (black bars) in the BNA2 family are shown as bar plots. Due to the high correlation between resistance to P2, P6 and P18, data are shown for only one of the three pathotypes. The y-axis represents the disease score from 1 (highly resistant) to 5 (highly susceptible). Genotypic classes are indicated on the x-axis. The number of individuals in each genotypic class is shown at the bottom of each bar. Error bars represent the standard deviation of the mean disease score of the genotypic class. **B**: Effects of allele dosage of SNP c1_4319_2 on resistance to P1 in the SaKa1 and BNA1 family.

Seven QRL for wart resistance were confirmed by SNP genotyping in the full BNA2 family (n = 133) (Table 2, Figure 2). Thirteen SolCAP SNPs were selected based on (i) *p*-value for linkage with wart resistance in the BNA2 subpopulation, (ii) parental allele dosage (preferentially simplex/nulliplex and simplex/simplex), (iii) representation of the major groups of co-segregating SNPs and (iv) putative linkage to previously mapped QTL for wart resistance (Figure 2). Pyrosequencing assays sensitive to the SNP allele dosage were designed for the selected SolCAP SNPs plus two additional SNPs in the locus PGSC0003DMG400006613, which contained SNP c2_33630 on chromosome XII (c2_33630_1 and c2_33630_2, see Additional file 3). The pyrosequencing assay designed for SNP c1_4319 on chromosome XI allowed to score two additional, new SNPs (c1_4319_2 and c1_4319_3), which were located six and nine nucleotides downstream of c1_4319 (see Additional file 3). The genotypes obtained by pyrosequencing were, with few exceptions, identical with the genotype calls from the SolCAP SNP array. Four SNP markers that were linked with wart resistance in the subset of 54 BNA2 individuals did not show significant linkage any more when genotyped in the full BNA2 family. Linkage of the remaining SNPs was confirmed, although the pathotype spectrum differed in several cases (Table 2, see Additional file 2). Most significantly linked with resistance to all four pathotypes were, based on chi-square values, the co-segregating SNPs c1_4319 and c1_4322 on chromosome XI, followed by c2_1106 and c2_35942 on chromosomes X and IV, respectively. All four SNPs scored on chromosome XII (c1_7770, c2_33630, c2_33630_1 and c2_33630_2) were linked to QRL specific for P2, P6 and P18. The segregation patterns of SNPs c2_33630_1 and c2_41100 were highly similar. SNP c2_2505 tagged the *RSe-Ib* locus on chromosome I but was linked with resistance to P1, whereas in the BNA1 and SaKa1 families, this locus conferred predominantly resistance to P2, P6 and P18 [5]. The effects on resistance of the most significant markers are shown in Figure 3A. Allele effects increased or decreased with the allele dosage.

Wart resistance alleles on chromosomes I (*RSe-Ib*) and XI (*RSe-XIa/Sen1*) were previously identified in the BNA1 and SaKa1 families [5]. To test whether these alleles also segregated in the BNA2 family, we genotyped a subset of 90 BNA2 genotypes for 55 SNPs in amplicons from markers linked with *RSe-Ib* (GP192, GP194 on chromosome I) and *RSe-XIa/Sen1* (GP125, GP259 and St_At5g17610 on chromosome XI, Figure 2). Of 24 SNPs scored on chromosome I only one (GP194_snp7) showed weak linkage with resistance to P18. This SNP was not significant in the BNA1 and SaKa1 families. Of 31 SNPs scored on chromosome XI, 12 were linked with resistance, either to all four pathotypes (2 SNPs), pathotypes P2, P6 and P18 (4 SNPs), pathotypes P1, P2 and P6 (1 SNP) or rather weakly to various singular or combinations of two pathotypes (5 SNPs) (Table 2). Six of the twelve SNPs had previously shown linkage with resistance to P1 in the SaKa1 family [5]. However, either the pathotype specificity differed between the families or the direction of the allele effect or both.

### New SNP markers linked with wart resistance loci in the BNA1 and SaKa1 families

The BNA1 and SaKa1 families were genotyped by pyrosequencing for SNPs linked with wart resistance loci in the full BNA2 family. With three exceptions, they did not show any linkage with wart QRL. The exceptions were SNPs c2_2505 on chromosome I, and c1_7770 and c2_33630_1 (co-segregating with c2_33630_2) on chromosome XII*.* SNP c2_2505 was linked with a QRL for all pathotypes in the SaKa1 family (Table 3) and with a QRL for P2, P6 and P18 in the BNA1 family (Table 4). The SNP allele *c2_2505_G* was linked in coupling phase with the QRL allele *2/6/18_b*, which increased susceptibility to pathotypes P2, P6 and P18 [5]. SNP c1_7770 detected a minor QRL for P1, P2 and P6 in the SaKa1 family on chromosome XII (Table 3). The allele *c1_7770_C* increased resistance in the SaKa1 family similar to the BNA2 family. SNPs c2_33630_1 and c2_33630_2 were linked with resistance to P1 in the SaKa1 family, although with inverted allele effects when compared to the BNA2 family (Table 3). In the BNA1 family, all three SNPs on chromosome XII were homozygous. Most interestingly, SNPs c1_4319_2 and c1_4319_3 (chromosome XI) that did not show any linkage with wart resistance in the BNA2 family (Table 2), tagged strong wart resistance alleles segregating in both SaKa1 and BNA1 families (Tables 3 and 4). SNP c1_4319_2 detected a major QRL for P1. The allele *c1_4319_2_G* linked with resistance (Figure 3B) was present in the resistant as well as in both susceptible parents of the BNA1 and SaKa1 families (simplex x simplex and simplex x duplex, Table 3) and was closely linked in coupling phase (3 recombinants in the SaKa1 family) with the *1_d* allele for resistance to pathotype P1 [5] inherited from the susceptible parent of the SaKa1 family. The allele SNP *c1_4319_3_T* was linked with a minor susceptibility allele for P1 in both families BNA1 and SaKa1 (Tables 3 and 4).

**Table 3:** **Novel markers linked with wart resistance in the SaKa1 family**

**Marker**	**Chr.**	**Alleles** ^**a**^	**Parental genotypes Pr**	**Parental genotypes Ps**	**P1, χ** ^**2**^	**P2, χ** ^**2**^	**P6, χ** ^**2**^	**P18, χ** ^**2**^
Solcap_c2_2505	I	*A↑/G*	*GAAA*	*AAAA*	7.26*^b^	11.98**	8.04*	6.80*
Solcap_c1_4319_2	XI	*G↑/A*	*GAAA*	*GAAA*	54.26***	ns^b^	ns	ns
Solcap_c1_4319_3	XI	*T/C↑*	*TCCC*	*CCCC*	5.51*	ns	ns	ns
Y1delATT	XI	*1/0↑*	0	1	6.20*	ns	ns	ns
Solcap_c1_7770	XII	*T/C↑*	*TTTT*	*TTCC*	6.34*	9.46**	7.99*	ns
Solcap_c2_33630_2 (= Solcap_c2_33630_1)	XII	*A/G↑*	*GGGG*	*GGGA*	9.64**	ns	ns	ns

^a^The allele linked with greater resistance is indicated by *↑*.

^b^ns: p > 0.05, *0.05 > p > 0.01, **0.01 > p > 0.001, ***p < 0.001.

**Table 4:** **Novel markers linked with wart resistance in the BNA1 family**

**Marker**	**Chr.**	**Alleles** ^**a**^	**Parental genotype Pr**	**Parental genotype Ps**	**P1, χ** ^**2**^	**P2, χ** ^**2**^	**P6, χ** ^**2**^	**P18, χ** ^**2**^
Solcap_c2_2505	I	*A↑/G*	*GAAA*	*AAAA*	ns^b^	9.83**^b^	10.46**	7.25*
Solcap_c1_4319_2	XI	*G↑/A*	*GAAA*	*GGAA*	13.01**	ns	ns	ns
Solcap_c1_4319_3	XI	*T/C↑*	*TCCC*	*CCCC*	7.55**	ns	ns	ns

^a^The allele linked with greater resistance is indicated by *↑*.

^b^ns: p > 0.05, *0.05 > p > 0.01, **0.01 > p > 0.001.

### The marker Y1delATT

Comparative amplicon sequencing in the *Y-1* candidate gene of resistant and susceptible clones of the BNA2 family identified a three base pair (ATT) insertion-deletion polymorphism in exon three of *Y-1* (see Additional file 3). The families BNA2, BNA1 and SaKa1 were genotyped for presence or absence of the ATT deletion using an allele specific PCR assay. The deletion was present in Pr-355, absent in Ps-354, segregated with a 1 : 1 ratio in the BNA2 family (Table 5) and was linked with high significance to resistance against all four pathotypes (Table 2). The *Y-1* locus physically maps between c1_4319/4322 and GP259 within 2 Mbp (Figure 2). Chi-square tests for independence between Y1delATT and c1_4319/4322 and GP259_snp7 were rejected with high confidence (p < 0.001), which indicated that these three markers were linked in coupling phase with a major wart resistance allele in parent Pr-355. Thirteen genotypes (9.8%) were recombinant between *Y1delATT_1* and *c1_4319_A/4322_T* and nine genotypes (10.2%) between *Y1delATT_1* and *GP259_snp7_G*. The segregation patterns of Y1delATT and the most significant SNP markers in the BNA2 progeny allowed the construction of parental haplotype models (Table 6). According to these models, haplotype H1 of Pr-355 included the major wart resistance allele. Haplotypes H2 and H3 of Ps-354 contributed minor positive and H4 present in both parents minor negative effects on resistance. In both the BNA1 and SaKa1 families, the Y1delATT marker was present only in the susceptible parent. In the SaKa1 family the marker was weakly linked with susceptibility to P1 (Table 3) and was not significant in the BNA1 family (not shown).

**Table 5:** **Genetic models for markers linked with wart resistance, expected segregation ratios and observed genotypes (n = nulliplex, s = simplex, d = duplex, t = triplex, q = quadruplex)**

**Marker allele**	**Chr.**	**Family**	**Model**	**Expected genotypes and ratios**	**Observed genotype numbers**	**χ** ^**2**^
*Solcap_c2_2505_A*	I	BNA2	simplex x triplex	1 s : 2 d : 1 t	4n, 23s, 55d, 43t, 5q^a^	7.6*^b^
*Solcap_c2_2505_G*	I	SaKa1	simplex x nulliplex	1 s : 1 n	49s, 68n, 2d^a^	3.1 ns^b^
*Solcap_c2_2505_G*	I	BNA1	simplex x nulliplex	1 s : 1 n	59s, 76n, 2d^a^	2.1 ns
*Solcap_c2_35942_A*	IV	BNA2	simplex x nulliplex	1 s : 1 n	65s, 67n	0.0 ns
*Solcap_c2_1106_G*	X	BNA2	simplex x nulliplex	1 s : 1 n	58s, 73n	1.7 ns
*Y1delATT_1*	XI	BNA2	simplex x nulliplex	1 s : 1 n	57s, 75n	2.4 ns
*Y1delATT_1*	XI	SaKa1	simplex x nulliplex	1 s : 1 n	62s, 57n	0.2 ns
*Solcap_c1_4319_A*	XI	BNA2	simplex x simplex	1 n : 2 s : 1 d	34n, 50s, 46d, 1t^a^	9.1**
*Solcap_c1_4319_2_G*	XI	SaKa1	simplex x simplex	1 n : 2 s : 1 d	36n, 61s, 21d, 1t^a^	3.9 ns
*Solcap_c1_4319_2_G*	XI	BNA1	simplex x duplex	1 n : 5 s : 5 d : 1 t	6n, 66s, 57d, 10t	4.1 ns
*Solcap_c1_4319_3_T*	XI	SaKa1	simplex x nulliplex	1 s : 1 n	63s, 56n	0.4 ns
*Solcap_c1_4319_3_T*	XI	BNA1	simplex x nulliplex	1 s : 1 n	72s, 68n	0.1 ns
*Solcap_c1_4322_T*	XI	BNA2	simplex x simplex	1 n : 2 s : 1 d	34n, 53s, 45d	6.9*
*GP259_snp7_G*	XI	BNA2	simplex x simplex	1 n : 2 s : 1 d	22n, 39s, 27d	1.7 ns
*St_At5g16710_snp3_C*	XI	BNA2	simplex x duplex	1 n : 5 s : 5 d : 1 t	5n, 38s, 36d, 8t	0.8 ns
*St_At5g16710_snp11_T*	XI	BNA2	simplex x duplex	1 n : 5 s : 5 d : 1 t	2n, 36s, 46d, 3t	8.9*
*Solcap_c1_7770_C*	XII	BNA2	simplex x duplex	1 n : 5 s : 5 d : 1 t	20n, 60s, 40d, 12t	12.0**
*Solcap_c1_7770_C*	XII	SaKa1	nulliplex x duplex	1 n : 4 s : 1 d	20n, 67s, 26d	3.7 ns
*Solcap_c2_33630_C*	XII	BNA2	duplex x duplex	1 n : 8 s : 18 d : 8 t : 1 q	4n, 19s, 104d, 0t, 3q	53.4***
*Solcap_c2_33630_2_A*	XII	BNA2	duplex x duplex	1 n : 8 s : 18 d : 8 t : 1 q	39n, 52s, 28d, 9t, 4q	392,6***
*Solcap_c2_33630_2_A* ( *Solcap_c2_33630_1_G*)	XII	SaKa1	simplex x nulliplex	1 s : 1 n	46s, 52n, 19d^a^	0.4 ns

^a^Exceptional genotypes present in the progeny were not considered in the goodness of fit test.

^b^ns: p > 0.05, *0.05 > p > 0.01, **0.01 > p > 0.001, ***p < 0.001.

**Table 6:** **Haplotype models for parents Ps-354 and Pr-355 at the** ***Sen1/RSe-XIa*** **locus**

	**Pr-355**				**Ps-354**			
**Locus**	**H1**	**H4**	**H5**	**H5**	**H2**	**H3**	**H4**	**H4**
*Solcap_c1_4319/4322*	AT*↑* ^a^	GC	GC	GC	AT*↑*	GC	GC	GC
*St_At5g16710_snp1/3/6*	AAT*↑*	TCA	AAT*↑*	AAT*↑*	AAT*↑*	AAT*↑*	TCA	TCA
*St_At5g16710_snp4*	G	G	G	G	G	C*↑*	G	G
*Y1delATT*	1*↑*	0	0	0	0	0	0	0
*GP259_snp7*	G*↑*	A	A	A	G*↑*	A	A	A
*GP259_snp16/17*	AC	AC	AC	AC	AC	GA*↑*	AC	AC

^a^The allele linked with greater resistance is indicated by *↑*.

### Marker segregation ratios

Genetic models for the most significant markers linked to wart resistance loci on chromosomes I, IV, X, XI and XII were deduced from the parental genotypes under the assumption of tetrasomic inheritance and tested for goodness of fit (Table 5). In several cases exceptional genotypes were observed in the progeny that were not compatible either with the parental genotypes or with tetrasomic inheritance, for example, nulliplex and quadruplex genotypes for SNP c2_2505 in the BNA2 family and duplex genotypes for SNP c2_33630_2 in the SaKa1 family. Such genotypes can result from scoring errors. However several of those genotypes were confirmed by two independent methods of SNP genotype calling, the SolCAP array as well as pyrosequencing. This indicated that some exceptional genotypes could result from chromatid segregation and double reduction [29]. When exceptional genotypes were excluded from the goodness of fit test (chi-square), the segregation ratios of the markers on chromosomes I, IV, X and XI were normal or slightly distorted. Marker segregation ratios on chromosome XII were highly distorted in the BNA2 family and did not fit the parental models.

### Marker combinations for wart resistance

**Figure 4:**
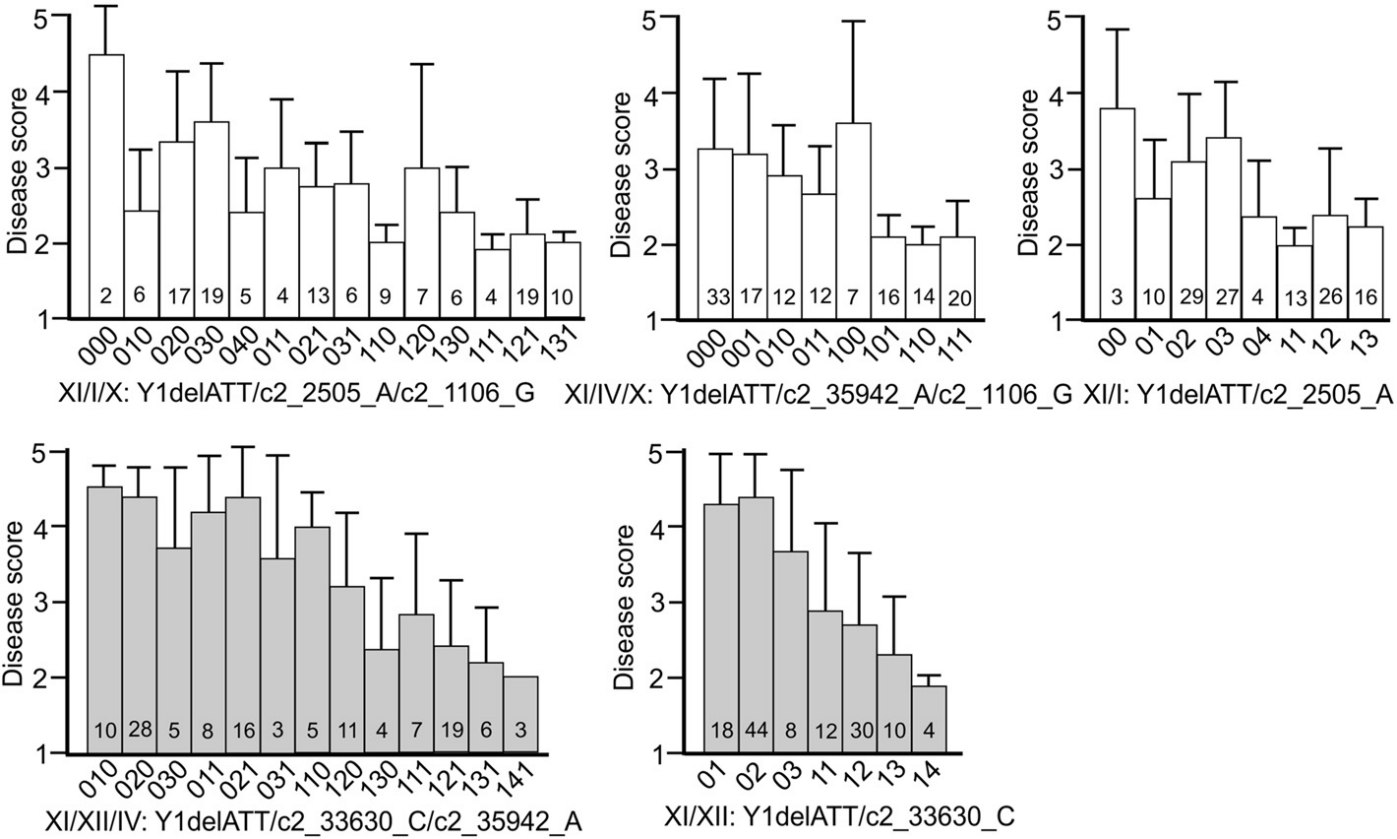
Effects of marker combinations on mean wart resistance. Effects of Y1delATT in combination with one and two SNP markers on resistance to P1 (white bars) and P2 (light grey bars, representative also for P6 and P18) in the BNA2 family are shown as bar plots. The y-axis represents the disease score from 1 (highly resistant) to 5 (highly susceptible). Presence/absence (1/0) of Y1delATT and SNP allele dosage between 0 and 4 is shown on the x-axis in the order of the markers as indicated below the x-axis. The number of individuals in each genotypic class is shown at the bottom of each bar. Error bars represent the standard deviation of the mean disease score of the genotypic class. Genotypic classes with only one individual are excluded.

Although markers Y1delATT and SNPs c1_4319/4322 tagged most effectively the major wart resistance locus on chromosome XI in the BNA2 family, one marker alone could not explain the observed phenotypic distributions (Figure 1). Combinations of Y1delATT and c1_4322 with one or two of the other highly significant markers were therefore tested with the Kruskal-Wallis test, in order to identify optimized marker combinations based on the highest chi-square values. For P1, the combination of *Y1delATT_1* with any of the alleles *c2_2505_A*, *c2_35942_A* or *c2_1106_G*, either alone or in pairs, performed better than any marker alone. For pathotypes P2, P6 and P18, the combination of *Y1delATT_1* with *c2_33630_C* alone, or with *c2_33630_C* plus any of the alleles *c2_2505_A, c2_35942_A* or *c2_1106_G*, performed better than any marker alone (see Additional file 4). Representative examples for the effects on wart resistance of combinations of *Y1delATT_1* with one or two additional markers are shown in Figure 4.

### Association with resistance to P1 in a variety panel

A collection of 83 varieties, plus 7 tetraploid and one diploid breeding clone was genotyped for the Y1delATT marker and eight SNPs most significantly linked with wart resistance loci in the BNA2, BNA1 and SaKa1 families. According to the available passport information, 28 varieties were susceptible to P1. The remaining 62 genotypes were resistant, eleven of which were also resistant to additional pathotypes. The variety ‘Flourball’ and breeding clones BRA 9089, MPI 44.1016/24 and MPI 50.247/2 were included in the panel as important sources of wart resistance according to Ross [3] as well as H80.577/1 (P3), the diploid source of *Sen1/RSe-XIa* [6](see Additional file 5). None of the markers tested showed a significant association with resistance to P1.

## Discussion

In order to identify loci controlling wart resistance in the BNA2 family, we pursued two strategies. First, bulked segregant analysis (BSA) [5,30] was performed using most SSR markers available in potato [22-24]. Second, a subset of BNA2 genotypes selected for phenotypic extremes was genotyped with the 8.3k SolCAP SNP genotyping array [25]. Using a subset instead of the full family was mainly motivated by the costs for custom genotyping. BSA resulted in two SSR markers that tagged the loci *RSe-IVb* and *RSe-VIa*, whereas genome wide SNP genotyping resulted in 67 SNPs that tagged thirteen main genomic regions, most prominent on chromosomes XI and XII. Subsequent validation of SolCAP SNPs in the full BNA2 family identified seven wart QRL (Figure 2). Genome wide SNP genotyping proved therefore superior to BSA with SSRs, mainly due to the increased marker density. However, the singular marker Y1delATT, which tagged most effectively the major wart resistance allele segregating in the BNA2 family, was developed based on a one amino acid deletion in the NB_LRR type candidate gene *Y-1*[18] that is closely linked with the *Sen1/RSe-XIa* locus. The SolCAP SNPs originated from the wart susceptible varieties Atlantic, Bintje, Kennebec and Shepody (http://www.europotato.org/menu.php?) and from varieties Snowden and Premier Russet with unknown wart resistance [25]. Most likely, none of these six genotypes carries major wart resistance alleles at the *Sen1/RSe-XIa* locus and therefore, SNP haplotypes specific for them were not present on the array. Surprisingly, none of the 31 additional SNPs scored on chromosome XI in the BNA2 family was linked, like Y1delATT, in coupling phase and simplex allele dosage with the major wart resistance allele. All these SNPs are located within a 3 Mbp genomic segment which includes *Y-1* and other members of the NB-LRR type gene family. The reason might be high recombination rates in this distal region of chromosome XI as observed in the BNA2 family. High recombination rates in the same region are also evident from the mapping experiment of Groth et al. [21], where the SSR marker StI018 was located at 50 cM on the linkage map of chromosome XI. On the physical map, this large genetic distance corresponds to only 6.4 Mbp (Figure 2). The opposite phenomenon was observed on chromosome XII, where the SNP markers tagging wart QRL were spread over the whole physical chromosome map (Figure 2). Several wart QRL might be present on chromosome XII. On the other hand, the groups of SNPs tightly linked in coupling phase and physically spreading across the central 40 Mbp might all tag the same locus (*RSe-XIIa*) in a central chromosomal region with low recombination rates.

Unlike the tetraploid families studied previously [5,21], resistance to *S. endobioticum* pathotypes 1, 2, 6 and 18 showed a bi-phasic distribution in the BNA2 family. This indicated the presence of a single, major resistance locus for each pathotype (Figure 1A). Moreover the strong correlation between resistance to the four pathotypes (Table 1) suggested that the same locus might confer resistance to all pathotypes. This locus was identified in the approximately 3 Mbp distal segment on the long arm of chromosome XI. This genomic position corresponds to the position of the *Sen1* locus on the genetic map, originally discovered by Hehl et al. [6] in diploid germ plasm. With one exception [20], *Sen1/RSe-XIa* has now been identified as the major contributor to wart resistance in all mapping studies performed so far including this paper [5,6,21]. Additional minor genes for wart resistance in the BNA2 family were located on chromosomes I, III, IV, VI, X and XII. The locus on chromosome I possibly corresponds to *RSe-Ib* discovered previously in the BNA1 and SaKa1 families [5], because all markers linked to wart resistance in this genomic segment map to the same 7 Mbp region. *RSe-VIa* was anchored by SSR marker StI004 to a distal position on the long arm of chromosome VI and likely corresponds to the QRL flanked by SSR markers StI015 and StI016 described by Groth et al. [21] (Figure 2). *RSe-IVb* was anchored to the approximately 3 Mbp most distal genomic region on the long arm of chromosome IV (Figure 2). The distal 3 Mbp correspond to recombination bins 78 to 105 on the high resolution chromosome IV genetic map of the diploid genotype RH [28], the wart susceptible parent of the population used for mapping the *Sen1-4* wart resistance gene [20]. Bridging AFLP markers between the genetic maps of genotypes RH and SH, the latter carrying the *Sen1-4* gene, place the 1 Mbp genomic segment including *Sen1-4* not further distal on the long arm of chromosome IV than RH recombination bin 50 (Herman van Eck, Wageningen University, personal communication). It is therefore unlikely that *RSe-IVb* corresponds to the *Sen1-4* locus. The QRL on chromosomes VII (*RSe-VIIa*) and VIII (*RSe-VIIIa*) identified by Groth et al. [21] were tagged by several SolCAP SNPs (Figure 2). However, the effects detected in the subset of the BNA2 family could not be validated in the full population. The QRL described by Groth et al. [21] on chromosomes I (*RSe-Ia*), II (*RSe-IIa*), X (*RSe-Xa*) and XI (*RSe-XIb*) were anchored via linked SSR markers to the physical chromosome maps (Figure 2). These QRL were not tagged by any marker in the BNA2 family, probably due to homozygosity or very small allele effects. Novel wart QRL described in this paper are *RSe-IIIa, RSe-IVb, RSeVIb, RSe-Xb and RSe-XIIa* (Figure 2). Taken all mapping studies together, resistance to *S. endobioticum* pathotypes 1, 2, 6 and 18 is controlled by the major locus *Sen1/RSe-XIa* and at least fifteen additional loci with smaller effects. The number of the genes controlling wart resistance might be even higher. The resolution of the genetic mapping studies does not allow to distinguish whether the effects at a particular QRL are controlled by a single gene or by several, physically linked genes such as clusters of NB-LRR type genes [17].

The genomic architecture of *S. endobioticum* resistance in the families BNA2, BNA1 and SaKa1 had in common the position of the *Sen1/RSe-XIa* and *RSe-Ib* loci but was otherwise different. The major resistance allele at the *Sen1/RSe-XIa* locus conferred resistance to all pathotypes in the BNA2 family, whereas it was specific for pathotype P1 in the BNA1 and SaKa1 families. A QRL for P2, P6 and P18 was mapped on chromosome I in the BNA1 and SaKa1 families (*RSe-Ib*), whereas in the BNA2 family, this locus affected only P1. This confirms the existence of multiple resistance alleles at the same locus within and between families [5]. Different QRL for P2, P6 and P18 were also identified on chromosomes IV, X, XI and XII. The differences between the crosses analyzed so far likely result from different historical sources of resistance to wart, four of which were genotyped for the markers linked to the main QRL. The origin of the Y1delATT marker might be the breeding clone MPI 50.247/2 mentioned by Ross [3] as source of resistance to pathotypes P1, P2 and P6. Information on resistance to P18 is not available for this clone. The Y1delATT marker was present exclusively in clone MPI 50.247/2 but not in the other three historical source genotypes tested (see Additional file 5). The frequency of the Y1delATT marker in a panel of European varieties was low. From ten genotypes with resistance to multiple *S. endobioticum* pathotypes only cvs Karolin and Kuba were clearly positive for the Y1delATT marker whilst the susceptible parents of the BNA1 and SaKa1 families and cvs Alegria, Arnika, Hansa and Ilona were also positive (see Additional file 5). No evidence was found for an association of the Y1delATT marker with resistance to P1 in European varieties. Association tests for resistance to P2, P6 and P18 were not feasible due to lack of phenotypic data for most varieties. The SNP allele *c1_4319_2_G* which tagged the major P1 specific resistance allele at the *Sen1/RSe-XIa* locus was common in the variety panel and was present in cv Flourball and breeding clone MPI 44.1016/24, both sources for resistance to P1. This marker failed to show an association with resistance to P1 like all other markers tested. Reasons for the lack of diagnostic power of the markers beyond the direct source genotype are multiple sources of resistance [2,3] and possibly high recombination rates around *Sen1/RSe-XIa*. Except *RSe-XIIa* (see above), the most relevant wart resistance loci map to distal chromosome ends with recombination hot spots [28]. Identification of the genes underlying the most important *RSe* loci and analysis of their allelic variation is advocated to obtain allele specific diagnostic DNA markers that are not compromised by recombination. The physical map (Figure 2) provides a starting point for positional cloning of genes for resistance to *S. endobioticum.*

Combinations of the major resistance allele at *Sen1/RSe-XIa* with one or two of the minor resistance alleles were sufficient to achieve high levels of resistance to *S. endobioticum* pathotypes 1, 2, 6 and 18 in the BNA2 family (Figure 4). Similarly, three to four alleles were required in the family analysed by Groth et al. [21]. The limited number of markers required opens up promising perspectives for marker-assisted selection of wart resistant cultivars. First, the major alleles present in a particular wart resistance source are tagged by genome wide SNP genotyping of a limited number of segregating progeny selected for high and low resistance, similar as demonstrated in this paper. Using the potato genome sequence [27,28] for anchoring the tagging SNPs, the major QRL are identified and anchored to the physical map of *RSe* loci as shown in Figure 2. Then, based on positional information, markers optimally linked with the *RSe* alleles present in a particular resistance source are selected, which can be used for marker-assisted selection in descendants of the resistance source.

## Conclusions

Genome wide SNP genotyping using the first generation 8.3k SolCAP array [25] was suitable for detecting and mapping the most relevant potato loci controlling resistance to *Synchytrium endobioticum*. A polymorphism in a NBS-LRR-type candidate gene provided the marker Y1delATT that tagged most effectively the major resistance locus in the BNA2 family. Genetic dissection with DNA-based markers in this and previous studies showed that *RSe-XIa/Sen1* on chromosome XI is the major locus for resistance to *Synchytrium endobioticum* in the potato genome. This locus has multiple resistance alleles with different pathotype specificities, which were introgressed from several historical sources in the European germ plasm pool of tetraploid potato. The effects on wart resistance of the major locus *RSe-XIa/Sen1* are genotype specific and are modified in a genotype specific manner by at least thirteen additional, independent loci with smaller effects that are located on most potato chromosomes. For high levels of resistance to *S. endobioticum* pathotypes 1, 2, 6 and 18 a combination of Y1delATT with one or two markers tagging secondary resistance alleles was sufficient. Resistance to the more recent *S. endobioticum* pathotypes 2, 6 and 18 is highly correlated, mostly controlled by the same loci and therefore largely tagged by the same markers. Depending on the resistance source, this can also apply to pathotype 1. The Y1delATT marker plus the SSR and SNP markers described in this paper and in previous papers [5,21] provide the basic information to aid marker-assisted breeding of wart resistant cultivars, starting from genetically characterized resistance sources. The markers are also useful for positional cloning of genes for resistance to potato wart.

## Methods

### Plant material

The tetraploid mapping population BNA2 consisted of 133 F1 progeny of a cross between a wart resistant (Pr-355) and a susceptible parent (Ps-354). Parent Pr-355 was resistant to *S. endobioticum* pathotypes P1, P2, P6 and P18 with mean resistance ratings of 1.86, 1.75, 1.90 and 1.82, respectively. Mean ratings of the susceptible parent Ps-354 were 2.35 (P1), 4.53 (P2), 5.00 (P6) and 4.40 (P18). The BNA2 family was generated by the breeding company Böhm-Nordkartoffel Agrarproduktion GbR (Ebstorf, Germany) and field propagated under the phytosanitary regimes for the production of seed tubers. The half sib families BNA1 (n = 141) and SaKa1 (n = 125) segregated for quantitative resistance to pathotypes P1, P2, P6 and P18 and have been used to map wart QRL [5]. Genomic DNA of varieties and breeding clones were from the collection of the Max-Planck-Institute for Plant Breeding Research, Cologne. The original plant material was either received from the IPK Gatersleben germplasm bank, External Branch North, Groß-Lüsewitz, or from the breeder of the variety [31] (see Additional file 5). Information on wart resistance of a given variety was retrieved from ‘The European Cultivated Potato Database’ (http://www.europotato.org/menu.php?) or from the ‘Beschreibende Sortenliste Kartoffeln 2012 (http://www.bundessortenamt.de/internet30/fileadmin/Files/PDF/bsl_kartoffeln_2012.pdf).

### Resistance evaluation

Resistance to *S. endobioticum* pathotypes 1, 2, 6 and 18 was evaluated as described [5]. Between 10 and 30 tubers per genotype and pathotype were inoculated and rated from 1 (completely resistant) to 5 (highly susceptible). Plants with scores 1 and 2 are rated highly resistant and resistant, respectively, with score 3 intermediate and plants with scores 4 and 5 are rated susceptible. Genotypes with clearly susceptible reactions in preliminary tests were finally evaluated with 10 tubers, whereas putative resistant and unclear genotypes were evaluated with up to 30 tubers. Mean scores were calculated from the individual scores of all infected tubers according to M = [a + 2b + 3c + 4d + 5e]/n, where a, b, c, d and e are the number of tubers scored with 1, 2, 3, 4 and 5, respectively, and n is the total number of scored tubers.

### DNA extraction

Total genomic DNA was isolated from 0.3 to 0.4g freeze dried leave tissue according to [32]. DNA concentration was estimated using a NanoDrop™ ND-1000 spectrophotometer (PeQLab Biotechnology GmbH, Germany). DNA quality was assessed on ethidium bromide containing agarose gels and by control PCRs using ubiquitin-specific primers UBQf (gaccatcactcttgaggttgag) and UBQr (aatggtgtctgagtctgagctctcgac), which generated a 300 base pair fragment using the annealing temperature 58**°**C.

### Bulked segregant analysis (BSA) using simple sequence repeat (SSR) markers

Based on the resistance evaluation, 12 genotypes with the highest resistance scores against all four wart pathotypes and 12 most susceptible genotypes were selected from the BNA2 family. A resistant and susceptible DNA bulk was constructed by mixing equal amounts of genomic DNA of the 12 resistant and 12 susceptible genotypes. The parents and the two bulks were screened with 195 SSR markers described in [22-24]. PCR reactions were performed in 25 μL buffer (20 Mm Tris HCl, pH 8.4, 1.5 Mm MgCl_2 ,_ 50 mM KCl) including 50 ng DNA template, 0.25 μM of each primer, 0.2 mM dNTPs and 0.2 Units *Taq* DNA polymerase (Invitrogen Life Technologies, Freiburg, Germany). PCR was carried out in a SensoQuest labcycler (SensoQuest Biomedicine Electronic, Germany). Cycling conditions were: 3 min initial denaturation at 94°C, followed by 35 cycles of 1 min denaturation at 94°C, 1 min annealing at the temperature reported in the literature and 1 min extension at 72°C, final extension for 10 min at 72°C. PCR products were confirmed by agarose gel electrophoresis. PCR products were separated on Spreadex gels (Elchrom Scientific, CH-6330 Cham, Switzerland) according to the supplier’s instructions. Markers showing qualitative or quantitative different banding patterns between the parents and between the bulks were re-screened in the parents and the 24 individual genotypes making up the bulks and finally in the parents and 94 randomly selected individuals of the BNA2 family.

### SNP genotyping using the 8.3k SolCAP potato SNP array and detection of linkage with resistance

Based on the resistance evaluation, 54 individuals were selected from the BNA2 family, which combined as much as possible the most resistant and most susceptible ratings for all four pathotypes, excluding most individuals with intermediate resistance scores. The parents and 54 progeny were genotyped for 8303 SNPs using the SolCAP potato genotyping array [25]. Custom genotyping was performed by the Department of Genomics, Life & Brain Center Bonn (Germany), on an Illumina iScan system using the Infinium assay. Genotypes *AAAA, AAAB, AABB, ABBB* or *BBBB* were called for 6286 SNPs and each individual using FitTetra software [33]. For linkage analysis genotypes were converted in numerical values 0, 1, 2, 3 and 4 corresponding to genotypes *AAAA, AAAB, AABB, ABBB* and *BBBB*. SNPs were tested for linkage with resistance using the Kruskal–Wallis test and RStudio software (version 0.97.318). The Bonferroni multiple comparisons test was used to correct for multiple testing. Significant *F* tests (*p* < 0.01) provided evidence for linkage between a SNP marker and wart resistance. Genotype calling of significant SNPs was manually confirmed using GenomeStudio software (version 2011.1, Illumina).

### SNP genotyping by pyrosequencing

Genomic sequences flanking the targeted SNP were retrieved from the potato genome browser (http://potato.plantbiology.msu.edu/cgi-bin/gbrowse/potato/) [27] and used for primer design. Amplicons between 100 and 700 base pairs were generated from approximately 50 ng genomic DNA in 25 μl buffer (Ampliqon) including 1.5 mM MgCl_2_, 0.2 mM dNTPs, 1 μM of each primer (one biotinylated) and 1U *Taq* polymerase (Peqlab). Sequences, primers and PCR conditions are shown in Additional file 3. Pyrosequencing [34] and SNP calling was performed using pyromark gold Q96 reagent kits (Qiagen, Hilden, Germany) and a pyrosequencer PSQ96™ MA (Biotage AB, Uppsala, Sweden) according to the suppliers protocols. Linkage between SNPs and wart resistance loci was detected using the Kruskal–Wallis test and SPSS 15.0 software (IBM).

### SNP genotyping by amplicon sequencing

Locus specific amplification and SNP calling in the amplicons of the markers GP192, GP194, GP125, GP259 and St_At5g17610 was performed as described [5]. Linkage with wart resistance loci was detected as described above.

### Allele specific amplification of the Y1delATT marker

The *Y-1* gene is one among a large cluster of NB-LRR type genes including homologues *Nl25* and *Nl27* of the tobacco *N* gene for resistance to *Tobacco Mosaic Virus* (TMV), which is closely linked to the *Sen1* locus on chromosome XI [6,18]. A *Y-1* allele characterized by a three base pair deletion ATT (see Additional file 3) in exon three was specifically amplified using the primers 5′CTGGTAGGGGAAAAAGAACGTG3′ (forward) and 5′GAAATCTTGAGTGAGCCATAGTC3′ (reverse). The PCR reaction was performed with the same conditions as for SNP genotyping by pyrosequencing. PCR cycling conditions were: 3 min initial denaturation at 94°C, followed by 35 cycles of 30 sec denaturation at 94°C, 30 sec annealing at 60°C and 1 min extension at 72°C, final extension for 5 min at 72°C. PCR products were detected by agarose gel electrophoresis.

### Genomic positions of SolCAP SNPs, RFLP and SSR markers

The genomic positions (version 4.03) of SolCAP SNPs were retrieved from the potato genome browser (http://potato.plantbiology.msu.edu/cgi-bin/gbrowse/potato/) [27]. DNA sequences of potato and tomato RFLP anchor markers were retrieved from the GABI primary database (http://www.gabipd.org/) [35]. Sequences flanking SSR markers located in genes were obtained via the corresponding GenBank accession [22,24]. Alternatively, SSR primer sequences were mapped directly to the genome with the expect threshold set to 1000. Sequences were mapped to the potato pseudomolecules (v4.03) using the BLAST sequence alignment tool at http://potato.plantbiology.msu.edu/integrated_searches.shtml.

## Additional files

Additional file 1:
**Gel pictures of SSR markers STM1002 and StI004.**
Additional file 2:
**Solcap SNPs, p values and SNP genotypes.**
Additional file 3:
**Marker sequences, primers and PCR conditions.**
Additional file 4:
**Chi-square values for marker combinations.**
Additional file 5:
**Variety panel, wart resistance passport data and marker genotypes.**

## Abbreviations

AFLP: Amplified fragment length polymorphism
BAC: Bacterial artificial chromosome
BSA: Bulked segregant analysis
Mbp: Mega base pairs
NB-LRR: Nucleotide binding-leucine rich repeat
PCR: Polymerase chain reaction
QRL: Quantitative resistance locus
RFLP: Restriction fragment length polymorphism
SNP: Single nucleotide polymorphism
SSR: Simple sequence repeat.
