# Supplementary material for: Genomic architecture of potato resistance to Synchytrium endobioticum disentangled using SSR markers and the 8.3k SolCAP SNP genotyping array
Source: BMC Genet. 2015 Apr 16;16:38. doi: 10.1186/s12863-015-0195-y (PMC4407358; doi:10.1186/s12863-015-0195-y)
Supplement: Additional file 1: — Gel pictures of SSR markers STM1002 and StI004. [file 12863_2015_195_MOESM1_ESM.pdf]

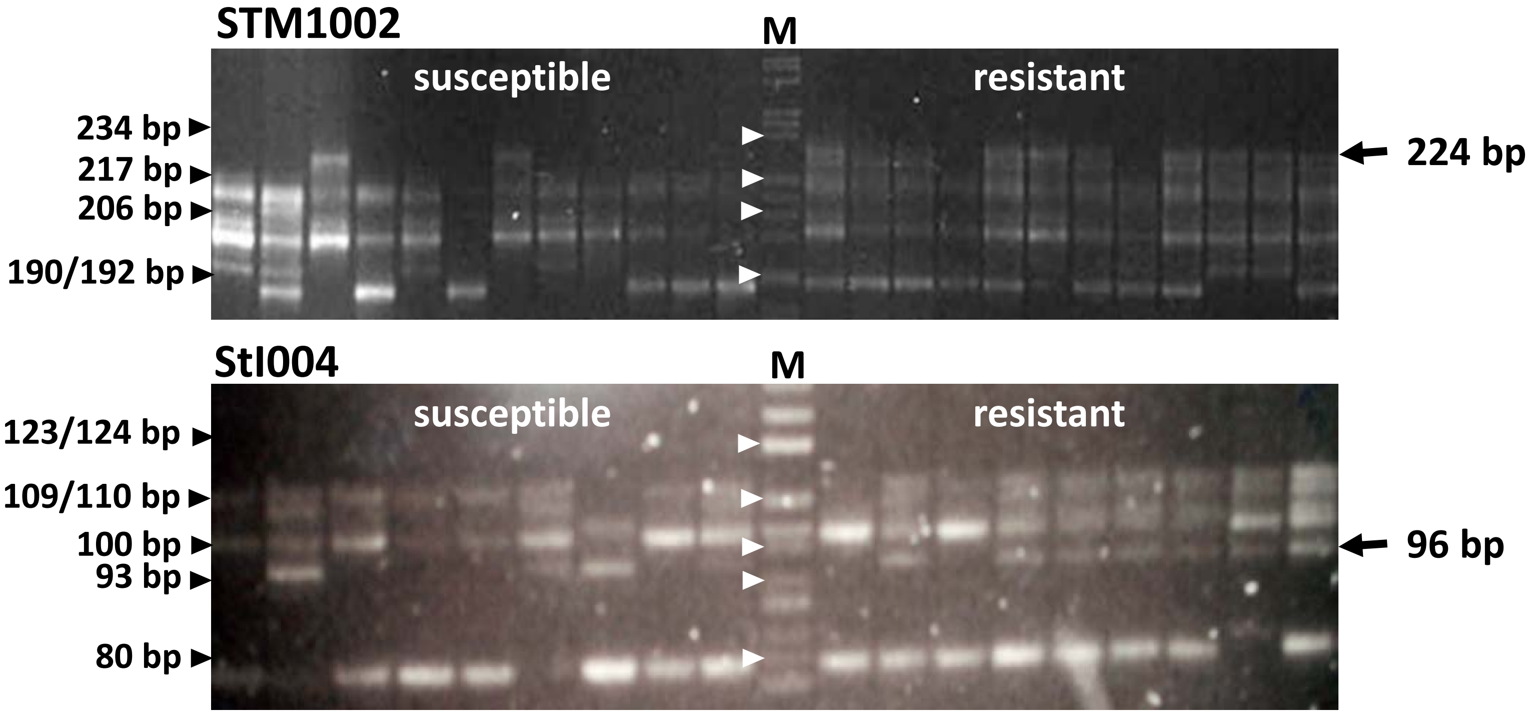

Additional file 1: Result of bulked segregant analysis (BSA) using SSR markers. Gel pictures (Spreadex, Elchrom Scientific, Switzerland) show the fragment patterns of markers STM1002 and StI004 in nine to twelve resistant and susceptible BNA2 genotypes used for constructing the DNA bulks. The sizes of the alleles linked to wart resistance loci are indicated on the right. M: size marker, fragment sizes are indicated on the left.
