# Supplementary material for: Genomic architecture of potato resistance to Synchytrium endobioticum disentangled using SSR markers and the 8.3k SolCAP SNP genotyping array
Source: BMC Genet. 2015 Apr 16;16:38. doi: 10.1186/s12863-015-0195-y (PMC4407358; doi:10.1186/s12863-015-0195-y)
Supplement: Additional file 3: — Marker sequences, primers and PCR conditions. [file 12863_2015_195_MOESM3_ESM.docx]

**Additional file 3**: Marker sequences, primers and PCR conditions. The SNP alleles as scored by pyrosequencing are indicated by red letters. In some cases the original Solcap SNP is defined by the complementary nucleotides, which are included in brackets and highlighted grey.

**solcap_snp_c2_2505** (chr01:77350867..77350888) PGSC0003DMG400024755

chr01:77350829..77350949

atgattttaaatatctctgtccttagtgcactctggtggcggaacagg**g/a**tacctcgctttatcagtgcaatagttataaacagtgtatttttgacggacccaacgaagtctcctgtattgt

c2_2505_forward: 5’ctgtccttagtgcactctggtg 3’

c2_2505_reverse(biotinylated): 5’CGTTGGGTCCGTCAAAAATACAC 3’

c2_2505_sequencing: 5‘CTCTGGTGGCGGAACAGG 3’

PCR conditions: 2min 94°C; 50 cycles of 93°C 30sec, 63°C 30sec, 72°C 30sec; 10min 72°C.

**solcap_snp_c1_6853** (chr03:42154541..42154562) PGSC0003DMG400014266

chr03:42154411..42154656

GAAGAGATAGGCTGAGTCCAGACTATGGTCGTGGCCGAGATCGGCCTAGTCCAGACTATGGTCGTGGTCGAGATCGGCCAAGTCCGGACTCTGGTCGTGGTCGAGATCGGCCAAGTCCTGACTATGGTCGTGGTCGAGAT**A/C**[T/G]GGCCTATTCCTGACTTTGGTCGTGGTAGAGATCGGCCAAGTGCTGACTTTGGTCGTGGTAGAGATCGGCTAAGTCCTGATTATGGTCGTGGCCCTAGTCGAAGTC

c1_6853_forward(biotinylated): 5’GAAGAGATAGGCTGAGTCCAGAC 3’

c1_6853_reverse: 5’GACTTCGACTAGGGCCACGAC 3’

c1_6853_sequencing: 5’CACGACCAAAGTCAGGAATAG 3’

PCR conditions: 2min 94°C; 50 cycles of 93°C 45sec, 64°C 45sec, 72°C 45sec; 10min 72°C.

**solcap_snp_c2_35942** (chr04:70878906..70878927) PGSC0003DMG400009868

chr04:70878871..70879032
CAATTGTCCAATTATGCCTGGAAAAAAATGGACATACAAATTTCA**A/G**ATGAAAGATCAGATTGGAAGTTTCTTCTATTTTCCTTCACTTTTCTTCCAGAAAGCTGCTGGAGGCTATGGTCCTATTCGAATTAACAATGTTGAGACTGTTCCTCTTCCATTTGCG

c2_35942_forward(biotinylated): 5’CAATTGTCCAATTATGCCTGG 3’ c2_35942_reverse: 5’CGCAAATGGAAGAGGAACAGTC 3’

c2_35942_sequencing: 5’CTTCCAATCTGATCTTTCAT 3’

PCR conditions: 2min 94°C; 50 cycles of 93°C 45sec, 53°C 45sec, 72°C 45sec; 10min 72°C.

**solcap_snp_c1_15965** (chr05:44622099..44622120) PGSC0003DMG400033879

chr05:44621867..44622223
GCAATGCAGACCCCGTGTTGTAGCCCCGATGAAGTGATATTCGGCCCTGATTTCAATCAATCCTTGTACTGTCATCTATTATGTGGTCTCATTCTACGTAACGAAGTTCAAGTTGTCTCATCTACCTTTGCTCATAGTATTGTCCATGCTTTTAGAAAATTCGAACAAGTATGGCAAGAACTTGTTGCAAATATAAGGGACGGAGTCCTTTCTAGTCGCGTAACTGTTCCTTCTATAAGATC**A/C**[T/G]GCAATGTCAAAATTACTCAAGCCTGATCCAGAACTGGCTGATGCTATTTATAACAAGTGTATTCGGTTAAGTAATTGGTACGGGTTGATACCTGAATTATTCCCAAACACCAGG

c1_15965_forward (biotinylated): 5’GCAATGCAGACCCCGTGTTGT 3’

c1_15965_reverse: 5’CCTGGTGTTTGGGAATAATTCA 3’

c1_15965_sequencing: 5’CTTGAGTAATTTTGACATTG 3’

PCR conditions: 2min 94°C; 50 cycles of 93°C 45sec, 58°C 45sec, 72°C 45sec; 10min 72°C.

**solcap_snp_c1_9224** (chr06:368277..368256)

PGSC0003DMG402007274

chr06:368336..368208

TAATTGCAGGTGGACACTAATCTGAAATCACCAGATGATTTTGTTACTGAGGGAGTGTCTGGTATCAGT**A/G**[T/C]AAACTTATGATGTTGCGCCGTACTCTGCTATCCTTCTTGAAGCAAAGCAATAATTACCG

c1_9224_forward (biotinylated): 5’TTGTTACTGAGGGAGTGTCTGG 3’

c1_9224_reverse: 5’CTTCAAGAAGGATAGCAGAGTACG 3’

c1_9224-sequencing: 5’TACGGCGCAACATCATAAGTTT 3’

PCR conditions: 2min 94°C; 50 cycles of 93°C 30sec, 61°C 30sec, 72°C 30sec; 10min 72°C.

**solcap_snp_c2_25250** (chr07:47642063..47642042) PGSC0003DMG400026161

chr07:47641948..47642298
CCCTTAGTAGTTCAAGTTGTTTCAATGCAGCCAATTCCTTGTCATCTATCATGCTTTCCTCCATTATGTCTAAGTGCAGTACCCGAAGTTGAGTCAGTGCAACGA**T/C**TTCACTCAGGCGACAAGCTTCTGTTGTTGCTGGACTCGGTATCTTGAACCCGTACAGCTCTTCAAGATTGGAAAGCCTCGAGAGACCTTGTGGTAAGCATGACAAGGATGGACAGCTTCCAACATCAAGAATTGCCAATCTTGGAAGTGTTGTGATTGATGTTGGTAGCATTTTCAGTTCCTTGCATTCTCTAAGGACTAGTATTTGAAGGCCCCATAACTTCCTTACGGAGTCTGAAATCTCGTC

c2_25250_forward (biotinylated): 5’CCCTTAGTAGTTCAAGTTGTTT 3’

c2_25250_reverse: 5’GACGAGATTTCAGACTCCG 3’

c2_25250-sequencing: 5’TGTCGCCTGAGTGAA 3’

PCR conditions: 2min 94°C; 50 cycles of 93°C 45sec, 55°C 45sec, 72°C 45sec; 10min 72°C.

**solcap_snp_c2_28588** (chr08:48364201..48364180) PGSC0003DMG400030865

chr08:48364128..48364310

ggaactggctccactccaagagattatagaatccatcatcgtcccgtactgaacaacgtgata**T/G**ttcaccgtgctgctaaagctggaactaaatagtgtaatgtaatgtaataaagtttctcgtggtcatccaaagtgtatagcctcagctgacaatttgaagtgtctatgaatgtataatggt

c2_28588_forward (biotinylated): 5’ACTGGCTCCACTCCAAGAGATT 3’

c2_28588_reverse: 5’CACTTTGGATGACCACGAGAAAC 3’

c2_28588-sequencing: 5’TAGCAGCACGGTGAA 3’

PCR conditions: 2min 94°C; 50 cycles of 93°C 30sec, 60°C 30sec, 72°C 30sec; 10min 72°C.

**solcap_snp_c2_1106** (chr10:2356805..2356784) PGSC0003DMG400021264

chr10:2356724..2356856
GGCCAAATTAAGCATGTGAGAGAAAGCTTTAGCAATGACAATGGAATCCCCTGGATCCAAACTTGTCAACA**G/C**ATTTCCAAGCTCCTCCAGCTTCTTTGGATCATGCTTTCCTTCGTACTCCGCAGAAAGTTCA

c2_1106_forward (biotinylated): 5’GCCAAATTAAGCATGTGAGA 3’

c2_1106_reverse: 5’TGAACTTTCTGCGGAGTACG 3’

c2_1106-sequencing: 5’GCTGGAGGAGCTTGGAAAT 3’

PCR conditions: 2min 94°C; 50 cycles of 93°C 30sec, 54°C 30sec, 72°C 30sec; 10min 72°C.

**solcap_snp_c1_4319** (chr11:939601..939580), **solcap_snp_c1_4322** (chr11:939934..939913)

PGSC0003DMG400013259

chr11:940185..939541

GCACGTGTCTCACCGAAGAACGTTCTTCTATTTGGAGCAGCTGATTATTAAGCACGATGCTGCTAATCGTGCTATAAAGATTCAACAGTTGGATCAGGGAATTGATTTCTTCTTTGGTCATAGAAGTCATGCTTTGAAGTTTGTGGATTTTGTCTGTAGGGTGGTACTTGTTAGGACCCGAAACGCCAAGCAACTCGCGTCTCATGATCATAAGAGCAATATCGTCAATTATAAGTACACATTCTCTG

c1_4322

TAGAAATCAGCCC[T/C]**A/G**GTTTGTCGTGAGGATCTGATATGTCTCCCTCCAAAGGTGGCAGCTAGTTTAGGAAATATCGGTCCTCTAGTGATCTGCACGAAAGTAAGCAACAGTATCTCTCTATTAGATCCGTTTACTCTGAGGCATTGTTTCCTAGATGCTGATCAGTACTGGAGGGCATCATTTAAGCCTTTACTATCTAGTAGACAGCTTGTCGAGTATGTGATTTTAGACGTTGATGTGGTTTCTGAAGAAGTTAATATTGGAGGCTCGAAGTATGTTTTAGCTGATATCCAAGTTGCTCGTG

c1_4319, c1_4319_2, c1_4319_3

TTTCTGATTTCGGGAAAAATGACACAATATTCTCCGTAAGAAC**A/G**CATCT**G/A**GG**C/T**CATCTTCTAGATGCTGGAGACTATGCCCTCGGTTATGATTT

c1_4322_forward(biotinylated): 5’GCACGTGTCTCACCGAAGAAC 3’

c1_4322_reverse: 5’CACATACTCGACAAGCTGTCTAC 3’

c1_44322_sequencing: 5’CATATCAGATCCTCACGACAAAC 3’

PCR conditions: 3min 94°C; 50 cycles of 94°C 30sec, 60°C 30sec, 72°C 60sec; 5min 72°C.

c1_4319_forward: 5’TGATTTCGGGAAAAATGACACAA 3’

c1_4319_reverse (biotinylated): 5’AAATCATAACCGAGGGCATAGTCT 3’

c1_4319_sequencing: 5’ACACAATATTCTCCGTAAGAAC 3’

PCR conditions: 3min 94°C; 50 cycles of 94°C 30sec, 60°C 30sec, 72°C 60sec; 5min 72°C.

**solcap_snp_c2_12276 (**chr11:8651914..8651893) PGSC0003DMG400009226

chr11: 8651634..8652052

CTAAACTTGAGTCACATCTCAGTGCAATACAGAAATCTGATATTGTATAAATTTACTTTCGATATTGAATAATTAGATACAATGGCTGACGAAAGTTATACATAAAGAAAAGTATGAGAATGAAAAACAAGAATACACAGCTAGAACCGAGCCCAACAGCGAGTGCATTTAGCCACACAATCACCTTTGCAATCAACATTGTAGCCTAAAACTTTTGGATTTCGAAGAAGCAAATTTCTCAGAGTTTT

c2_12277 c2_12276

ACCTTCAATTCCCCATTG**T/C**TTT**T/G**[A/C]CCAGAATTTCGACATTGGTTTTCATTTCATCTTCAAGGCTACATCCAAGAACTTCTGGGAATTTCTTGACAAGTTTAGTGAGATCATCATCAGTAAGGCTTAAACTCCTTAGATAATCCAGTATCTCATTCACAACTTCTAGTCGTCC

c2_12276_forward:5’CTAAACTTGAGTCACATCTCAGTG 3’

c2_12276_reverse(biotinylated):5’GGACGACTAGAAGTTGTGAATG 3’

c2_12276_sequencing:5’GTTTTACCTTCAATTCCCCATT 3’

PCR conditions: 3min 94°C; 50 cycles of 94°C 30sec, 60°C 30sec, 72°C 60sec; 5min 72°C.

**solcap_snp_c1_7770** (chr12:6701041..6701020), not annotated

chr12: 6700934..6701112

ctcttgaggagctggaggaaatcgtccttggaggaatcagccataacctctgaaatcttcaaagttgagagaattcagtgattcgttggtagtgtgg**A/G**[T/C]gccgatgaagattgatcaagttctccgctgttgcagaatgttctagaagaaatcgatcttgcagaggaagcttcgattgaa

c1_7770_forward: 5’CTCTTGAGGAGCTGGAGGAAATC 3’

c1_7770_reverse (biotinylated): 5’GCAACAGCGGAGAACTTGATCAA 3’

c1_7770_sequencing: 5’ttcgttggtagtgtgg 3’

PCR conditions: 2min 94°C; 50 cycles of 93°C 30sec, 63°C 30sec, 72°C 30sec; 10min 72°C.

**PGSC0003DMG400006613** (chr12:45977400..45970400)

chr12:45977090..45976461

ACAAAACAAAGCGTACTAAGGAAATTTTACATTTAATAACCAACATTAGTAACAATGTCGTA

c2_33630_1

AAGACATGAGAATACATACCCTAAGCTGCTGAAGTTGTGCTTCTGT**A/G**AAGCCCCCATCTTGTTCTGTTCCTGATTTTGCCATTAGTTGAACAACCCAGTTTGCAGCTTCACTATAGTCTTGTTGTGTAGCCCGGAAGAATTGCAGCTTTAGGTTCTGAATGAAGGACAGGGCAAGCAGACCTTCTCTATCAAAGTAAGGATGAGCTAAAGCTGTCTTTGCACTAATTCTTTGGCGTGCTTTGAAACGTACCATAGATGTTAATAGCTCCCAACCTATTCCACCATCCAAATCTAACAACTCAAAGCCTTTCCTAAGTTCAGGGCCAGCACGAGGCTCTACACTCTGTCTCCACGAAACTAAATCATAGTC

c2_33630_2

ACATCTCTTCAGTTGTCGGTTGAATTGAATGAGACTGTT**G/A**TCACTGCGTAATCCTGGGAATGCCTA

Forward (biotinylated):5’ACAAAACAAAGCGTACTAAGGA 3’

Reverse:5’TAGGCATTCCCAGGATTACGCA 3’

c2_33630_1_Sequencing: 5’GAACAGAACAAGATGGGGGCTT 3’

c2_33630_2_Sequencing: 5’CATTCCCAGGATTACGCAGTGA 3’

PCR conditions: 3min 94°C; 50 cycles of 94°C 30sec, 60°C 30sec, 72°C 60sec; 5min 72°C.

**solcap_snp_c2_33630** (chr12:45970808..45970787) PGSC0003DMG400006613

chr12: 45970629..45970918

GTCGATACGATTGAAGAAACAATGTAATTTTTCAATTTGCGGTAGCTTTGTACATGATCTTTTTGTTGGAGTTGGGGTAGGACTACCCTGTACTGTAATGGAGTGTGGTGATATAATTTATAGAAGCACACTACCCAAGTCAAATGGGATCACAGTGACTGTTCCTGGG**A/G**TTATTTTGGCTTTGGGTACCCTCTCTTACCTCTGGGCTACTCCTGGTGTTGCTCCTGGTTTCTTTGATATGTTTGTTCTTGCCTTTCTTGAAAGATTCTTTCGACCTATTTATAAGAAGG

c2_33630_forward: 5’GTCGATACGATTGAAGAAACAATG 3’

c2_33630_reverse (biotinylated): 5’CCTTCTTATAAATAGGTCGAAAG 3’

c2_33630_sequencing: 5’ATCACAGTGACTGTTCCTGGG 3’

PCR conditions: 3min 94°C; 50 cycles of 94°C 30sec, 60°C 30sec, 72°C 60sec; 5min 72°C.

**Y1delATT** (Vidal et al. 2002, MPMI 15:717) AJ300266 (1835..2674)

ATACTCTCATCTAAATTTGATGGTGCTTGTTTCCTTCCGGACAATAAAGAAAACAAGTATGAAATACATTCTCTGCAAAGTATCCTTCTCTCTAAACTGGTAGGGGAAAAAGAAA**ATT**GTGTGCATGATAAGGAGGACGGGAGGCACCTGATGGCTCGTAGACTTCGTTTGAAGAAAGTTCTAGTTGTGCTTGATAACATAGATCATGAAGACCAATTGAAATACCTAGCAGGGGATCTTGGTTGGTTTGGCAATGGCACCAGAATTATTGCAACAACGAGAGACAAGCATTTCATTCGGAAAAATGATGCCGTATATCCTGTGACTACACTACTTGAACATGATGCTGTTCAGTTgttcaaccaatatgctttcaaaaatgaagttccagataagtgtttcgaggagataacgttggaggtagtaagtcatgctgaaggccttcctttagccctgaaagtgtggggttcttccttacataagaaggatatacatgtgtggaggagtgctgttgatcgaataaagaggaaccctagttcaaaagttgttgaaaacctcaaagtaagttatgatgggttggagcgcgaagatcaagagatatttctagatattgcatgcttcttaagagggagaaaacaaaccgagatcaagcaaattcttgagagctgtgattttggagctgatgacggattgagggtgctgattgacaagtctcttgtgttcatctctgaatatgatacgattcaaatgcatgacttaatacaagagatgggtaaatacatagtgacaatgcaaaaggatcggggagaagtcactagactatggctcactcaagatttc

Y1delATT_forward(allele specific): 5’CTGGTAGGGGAAAAAGAACGTG 3’

Y1_forward (amplicon sequencing): 5’GGACAATAAAGAAAACAAGTATG 3’

Y1delATT_reverse (amplicon sequencing and allele specific): 5’GAAATCTTGAGTGAGCCATAGTC 3’

PCR conditions: 3min 94°C; 35 cycles of 94°C 30sec, 60°C 30sec, 72°C 60sec; 5min 72°C.
