# Supplementary material for: Genomic architecture of potato resistance to Synchytrium endobioticum disentangled using SSR markers and the 8.3k SolCAP SNP genotyping array
Source: BMC Genet. 2015 Apr 16;16:38. doi: 10.1186/s12863-015-0195-y (PMC4407358; doi:10.1186/s12863-015-0195-y)
Supplement: Additional file 4: — Chi-square values for marker combinations. [file 12863_2015_195_MOESM4_ESM.docx]

**Additional file 4:** Marker combinations for increased resistance to *S. endobioticum*. Chi-square values for the marker combinations shown in Figure 4 are underlined. p values were < 0.001, except where indicated otherwise by * (p < 0.05) or ** (p < 0.01).

| Marker combinations | Chr. no. | P1, χ^2^ | P2, χ^2^ | P6, χ^2^ | P18, χ^2^ |
| --- | --- | --- | --- | --- | --- |
| *Y1delATT* and *c2_2505_A* | XI/I | 46.67 | 52.69 | 47.79 | 50.66 |
| *Y1delATT* and *c2_35942_A* | XI/IV | 39.53 | 53.97 | 50.57 | 49.37 |
| *Y1delATT* and *c2_1106_G* | XI/X | 37.93 | 51.47 | 45.93 | 45.20 |
| *Y1delATT* and *c1_4322_T* | XI/XI | 35.40 | 58.46 | 52.38 | 47.55 |
| *Y1delATT* and *GP259_snp7_G* | XI/XI | 36.84 | 38.33 | 32.76 | 40.09 |
| *Y1delATT* and *St_At5g16710_snp11_T* | XI/XI/ | 35.78 | 38.46 | 34.12 | 40.69 |
| *Y1delATT* and *c2_33630_C* | XI/XII | 36.01 | 63.83 | 59.26 | 58.13 |
| *Y1delATT* and *c2_33630_1_A* | XI/XII | 31.99 | 51.60 | 49.66 | 52.55 |
| *Y1delATT* and *c2_33630_2_A* | XI/XII | 34.30 | 57.10 | 53.78 | 54.62 |
| *Y1delATT* and *c1_7770_C* | XI/XII | 35.89 | 59.12 | 51.85 | 59.35 |
| *Y1delATT* and *c2_35942_A* and *c2_1106_G* | XI/IV/X | 49.56 | 56.86 | 53.04 | 57.54 |
| *Y1delATT* and *c2_2505_A* and *c2_1106_G* | XI/I/X | 58.69 | 59.36 | 55.97 | 58.09 |
| *Y1delATT* and *c2_35942_A* and *c2_2505_A* | XI/IV/I | 50.92 | 54.16 | 50.34 | 52.76 |
| *Y1delATT* and *c2_33630_C* and *c2_35942_A* | XI/XII/IV | 45.75 | 70.63 | 65.04 | 64.28 |
| *Y1delATT* and *c2_33630_C* and *c2_2505_A* | XI/XII/I | 51.55** | 63.33 | 66.86 | 63.36 |
| *Y1delATT* and *c2_33630_C* and *c2_1106_G* | XI/XII/X | 43.73 | 71.93 | 64.60 | 62.38 |
| *c1_4322_T* and *c2_2505_A* | XI/I | 46.60 | 46.15 | 56.84 | 34.88 |
| *c1_4322_T* and *c2_35942_A* | XI/IV | 30.12 | 42.37 | 49.54 | 28.41 |
| *c1_4322_T* and *c2_1106_G* | XI/X | 31.16 | 41.91 | 44.93 | 28.60 |
| *c1_4322_T* and *c2_33630_C* | XI/XII | 21.83 | 47.18 | 53.96 | 32.73 |
| *c1_4322_T* and *c2_33630_1_A* | XI/XII | 18.80* | 33.88 | 42.18 | 29.22 |
| *c1_4322_T* and *c2_33630_2_A* | XI/XII | 24.21** | 46.16 | 54.34 | 36.83 |
| *c1_4322_T* and *c1_7770_C* | XI/XII | 32.75** | 51.01 | 52.97 | 43.71 |
| *c2_35942_A* and *c2_1106_G* | IV/X | 28.81 | 23.12 | 20.29 | 26.22 |
